# An Account of Some Experiments Made with a View to Ascertain the Comparative Quantities of Amylaceous Matter, Yielded by the Different Vegetables Most Commonly in Use in the West India Islands

**Published:** 1797

**Authors:** James Clark

**Affiliations:** Physician in Dominica, and Fellow of the College of Physicians and Royal Society of Edinburgh


XXVI. *An Account of ſome Experiments made with a view to aſcertain the comparative Quantities of amylaceous Matter, yielded by the different Vegetables moſt commonly in uſe in the Weſt India Iſlands. Communicated in a Letter to Dr.* Simmons, *by* James Clark, *M. D. F. R. S. Edin.*

STARCH, or the amylaceous matter of vegetables, has juſtly been conſidered as their moſt alimentary or nutritive part. By the advice of my friend, Dr. Wright, of Edinburgh, I un-

I undertook to make ſome experiments with a view to aſcertain the comparative quantities of this ſubſtance in the roots or other parts of vegetables moſt commonly in uſe in the Weſt-India iſlands.

I began with the roots of *Maranta arundinacea*, commonly called arrow-root.

A quantity of theſe roots, weighing four pounds avoirdupois, being firſt well ſcraped and cleaned, was grated or raſped, and about a gallon of water poured upon it and ſtrained through a clean cloth. It was then left to ſettle for ſome time, and the water being poured off, freſh water was added, and ſo on, repeatedly, till it was quite clean. It was then expoſed to the ſun, to dry, for ſome days; and when perfectly dry, it yielded five ounces of very fine white ſtarch. Similar quantities of other roots, &c. were treated exactly in the ſame manner, (care being taken to ſelect for the experiments only ſuch as were full grown) and after three trials of each, the medium products were as follows;

| | ozs. | drs. |
|---|---|---|
| 1. *Maranta arundinacea*, (arrow-root) - - | 5 | 0 |
| 2. *Jatropha Janipha*, (ſweet Caſſada) not poiſonous, - | 13 | 6 |

3. *Jatropha*

| | | ozs. | drs. |
|---|---|---|---|
| 3. *Jatropha Manihot*, (common bitter Caſſada,) the water of which is poiſonous, | - | 10 | 6 |
| 4. *Dioſcorea triphylla* (couch-couch, or yampee) | - | 5 | 2 |
| 5. *Dioſcorea bulbifera*, (Guinea yam) | - | 8 | 0 |
| 6. *Convolvulus Batatas*, (Weſt-India ſweet potatoe) | - | 8 | 0 |
| 7. *Arum eſculentum*, (Eddoes, white and yellow Tanniers, Malingas, or Coccos) | - | 11 | 0 |
| 8. *Muſa paradiſiaca*, (plantanes) full grown, (but not ripe,) peeled and grated, | - | 10 | 2 |
| 9. And by way of compariſon, I tried *Solanum tuberoſum*, (potatoes) procured here from Ireland, | - | 6 | 2 |

Four pounds of ſuperfine Baltimore flour yielded two pounds of indifferent ſtarch; but this was not a fair trial, as the huſks ought to have been taken into the account; but no wheat is ever brought to the Weſt Indies. On each of theſe I ſhall beg leave to add a few remarks.

1. The ſtarch of the *Maranta arundinacea*, or arrow-root, is uſed as a light nouriſhment for those affected with diseases of the breast or bowels, particularly in dysentery, lienteria, &c. A tea spoonful of it mixed with a little cold water, forms, with the addition of half a pint of boiling water, a mucilaginous drink, to which either milk or wine is added as may be found necessary; and it is rendered grateful to the palate and stomach by the addition of sugar and nutmeg. Administered in this way, it answers the purposes of drink and nourishment, and often removes diseases proceeding from debility of the stomach and bowels, without the aid of medicines. It is never used as food for healthy people.

2. 3. The starch of the *Jatropha Janipha*, or sweet Cassada, answers the same purpose as that of the *Maranta arundinacea* in every respect, and both have been found preferable to sago, in all cases where a lubricating, easily digested diet was indicated.

The starch of the *Jatropha Manihot* is equally nourishing as that of the *Jatropha Janipha*, but care must be taken to dry it very well, as the juice is poisonous. By turning this starch with a flat piece of wood on a plate of iron (called a farine pan) well heated, the tapioca is made, that has been so much recommended for its nutritive tritive and reftorative qualities all over Europe for many years paft. I have made tapioca in this way, and ufed it; but the ftarch, when thoroughly dried, anfwers equally well, and is prepared with lefs trouble.

The roots of this poifonous plant, when full grown, which is near a year after its being planted, are wafhed, and grated and turned, with a wooden fpatula on a hot plate of iron till quite dry, by which operation very fmall grains called *farine* are formed. This is ufed as bread, by the planters and their negroes, in thefe iflands, and it is found to be extremely nourifhing, and a very wholefome food. Cakes are alfo made of this farinaceous fubftance, upon the fame pan or plate of iron, which are eaten by all defcriptions of people, and preferred by many to bread made of flour.

The *Jatropha Janipha* is not in common ufe; the chief reafon of this is to prevent miftakes, as many negroes have been poifoned by roafting and eating the other fpecies inftead of this. From the great quantity of ftarch that it yields, it appears, however, to be the moft nourifhing of the two, but it is not fo productive, having only a few fmall roots. It is diftinguifhed from the bitter or poifonous fort by a ftrong ligneous fibre in the middle middle of each root, and is roasted or boiled when used at table, but never made into *farine*.

4. The roots of the *Dioscorea triphylla*, or couch-couch, are a very delicate food when roasted or boiled, and are thought to be equal to Irish potatoes; but they are used only by the white inhabitants, not being so productive as the other species of yams.

5. 6. 7. The *Dioscorea bulbifera*, or Guinea yam; *Convolvulus Batatas*, or sweet potatoe; and *Arum esculentum*, eddoes or coccos, (called *malingas* by the French;) are the chief support of many planters as well as of the negroes in the West Indies. The two first contain equal quantities of starch, and they are all three found to be remarkably nourishing when well boiled or roasted. The *arum esculentum*, or eddoe, makes the finest starch; and the roots boiled into a thick soup, and given to negroes, or others, affected with dysenteric complaints, prove to be a very salutary diet for them.

8. The *Musa paradisiaca*, or plantane tree, yields a fruit, which, when cut down green, and boiled or roasted, answers every purpose of bread, and may be styled with great propriety, the bread fruit of the West Indies.

It is a hearty food for hard-working people, and when allowed to ripen, is a pleaſant and grateful fruit. It contains a great proportion of ſtarch, a tea ſpoonful of which made, with half a pint of boiling water, a thick mucilaginous kind of gruel, which had ſomewhat of the flavour of the ripe fruit, and was very grateful to the palate.

I tried to make ſtarch from the *Muſa Sapientum*, or Banana, when pulled green and full grown; but the water which was poured upon it became glutinous, and afterwards fermented and turned into vinegar. I tried to get ſtarch from other fruits, but to no purpoſe. The common yam, when grated before it was full grown, turned the water into a mucilaginous ſtate from which no ſtarch was depoſited; this alſo fermented and became vinegar. The *phaſeolus*, ſo much in vogue in the iſlands of St. Domingo and Martinico ſome years ago, was planted and tried here, but the roots were very fibrous, or rather ſticky, and did not contain much farinaceous matter, on which account the cultivation of this Plant was given up.

Theſe are the chief roots made uſe of in this and moſt of the other Weſt-India Iſlands; but there is a great variety of the leguminous claſs, which make a conſiderable part of the diet of the

2 inhabiinhabitants. Theſe alſo contain ſtarch, and are looked upon to be very nutritious. The ſaccharine matter which all fruits and vegetables contain, in a greater or leſs degree, is alſo very nouriſhing. The cane plant contains this ſalt or matter in the greateſt proportion, and the nutritive quality of its juice is unqueſtionably very great; as the negroes in crop time, on ſugar plantations, are obſerved to be conſtantly fat and healthy, and their chief nouriſhment, during that time, is known to be this juice raw or a little boiled.

The mucilaginous part of vegetables, particularly of the *hibiſcus eſculentus*, or okra, as well as of many other ſucculent plants, alſo contributes a little towards the nouriſhment and ſupport of the inhabitants of theſe iſlands. All the Weſt-India roots I have enumerated, when boiled or roaſted, and pounded, may eaſily be converted into bread, if mixed with one half or two thirds the quantity of flour, and afterwards fermented with leven.

Whether the ſtarch produced from the Iriſh potatoes will be conſidered as a fair comparative trial, I ſhall not take upon me to determine. I took ſome pains, however, to ſelect the beſt that could be procured for theſe experiments, but found that the ſame weight yielded leſs ſtarch on every trial than the Weſt-India roots, thoſe of the *Maranta arundinacea*, and *Dioſcorea triphylla* excepted. Perhaps this may be attributed to their having been long kept; for there can be no doubt of potatoes being poſſeſſed of extraordinary nutritive qualities: I cannot help thinking, however, that the inhabitants of theſe iſlands are at no loſs for ſubſtitutes equally wholeſome, and as nouriſhing.

*Dominica,*
*May* 21, 1796.

